# Evidence that miR‐146a attenuates aging‐ and trauma‐induced osteoarthritis by inhibiting Notch1, IL‐6, and IL‐1 mediated catabolism

**DOI:** 10.1111/acel.12752

**Published:** 2018-03-24

**Authors:** Ying‐Jie Guan, Jing Li, Xu Yang, Shaohua Du, Jing Ding, Yun Gao, Ying Zhang, Kun Yang, Qian Chen

**Affiliations:** ^1^ Bone and Joint Research Center The First Affiliated Hospital and Frontier Institute of Science and Technology Xi'an JiaoTong University Xi'an China; ^2^ Cell and Molecular Biology Laboratory Department of Orthopaedics Alpert Medical School of Brown University/Rhode Island Hospital Providence RI USA; ^3^ Department of Orthopaedics Affiliated Hospital of Medical College of Qingdao University Qingdao China

**Keywords:** aging, cartilage, inflammation, miR‐146a, Notch1, osteoarthritis

## Abstract

Primary osteoarthritis (OA) is associated with aging, while post‐traumatic OA (PTOA) is associated with mechanical injury and inflammation. It is not clear whether the two types of osteoarthritis share common mechanisms. We found that miR‐146a, a microRNA‐associated with inflammation, is activated by cyclic load in the physiological range but suppressed by mechanical overload in human articular chondrocytes. Furthermore, miR‐146a expression is decreased in the OA lesions of human articular cartilage. To understand the role of miR‐146a in osteoarthritis, we systemically characterized mice in which miR‐146a is either deficient in whole body or overexpressed in chondrogenic cells specifically. miR‐146a‐deficient mice develop early onset of OA characterized by cartilage degeneration, synovitis, and osteophytes. Conversely, miR‐146a chondrogenic overexpressing mice are resistant to aging‐associated OA. Loss of miR‐146a exacerbates articular cartilage degeneration during PTOA, while chondrogenic overexpression of miR‐146a inhibits PTOA. Thus, miR‐146a inhibits both OA and PTOA in mice, suggesting a common protective mechanism initiated by miR‐146a. miR‐146a suppresses IL‐1β of catabolic factors, and we provide evidence that miR‐146a directly inhibits Notch1 expression. Therefore, such inhibition of Notch1 may explain suppression of inflammatory mediators by miR‐146a. Chondrogenic overexpression of miR‐146a or intra‐articular administration of a Notch1 inhibitor alleviates IL‐1β‐induced catabolism and rescues joint degeneration in miR‐146a‐deficient mice, suggesting that miR‐146a is sufficient to protect OA pathogenesis by inhibiting Notch signaling in the joint. Thus, miR‐146a may be used to counter both aging‐associated OA and mechanical injury‐/inflammation‐induced PTOA.

## 1 INTRODUCTION

Osteoarthritis (OA), the most prevalent aging‐related joint disease worldwide, is a major cause of disability that carries an extremely high socioeconomic burden (Hunter, Schofield & Callander, 2014). Osteoarthritis usually manifests as degeneration of articular cartilage, but other joint tissues, such as subchondral bone and the synovial membranes, are also affected. These alterations are responsible for pain, joint failure, and loss of joint architectural integrity. Current treatments for OA are limited to pain management and, in the late phase of the disease process, joint‐replacement surgery (Loeser, Goldring, Scanzello & Goldring, 2012). There are two major forms of OA. While primary OA occurs in response to slow but steady increase in inflammation during aging, secondary OA is trigged by trauma‐induced mechanical damage. Thus, mechanical overload and inflammation are two major extrinsic factors causing OA (Lotz & Kraus, 2010). Most of these disease outcomes are associated with abnormal differentiation of chondrocytes coupled with an imbalance in the turnover of cartilaginous extracellular matrix (ECM). Although genetic factors have been implicated in the pathogenesis of OA, the epigenetic pathways that precisely regulate the catabolic or anabolic responses and the balance of these processes are just beginning to be understood (Reynard & Loughlin, 2013). Understanding the molecular mechanisms to maintain joint cartilage homeostasis will be extremely important for developing future disease‐modifying OA drugs (DMOADs).

MicroRNAs (miRNAs) have emerged as important modulators in development, tissue homeostasis, and diseases via epigenetic pathways (Jovanovic & Hengartner, 2006). The miRNA–RISC complex mediates the degradation of specific mRNA targets and/or the repression of mRNA translation via interactions with the 3′ untranslated regions (UTRs) that are partially sequence‐specific (Jovanovic & Hengartner, 2006). Postnatal Drosha deficiency induces articular chondrocyte death and causes a mild OA‐like pathology (Kobayashi et al., 2015), and miR‐140 is involved in the pathogenesis of osteoarthritis by regulating ADAMTS5 (Araldi & Schipani, 2010). These findings suggest that miRNAs play a critical role in cartilage homeostasis. Recent studies have revealed that miR‐146 is primarily involved in the regulation of inflammation and other processes that function in the innate immune system (Sonkoly, Stahle & Pivarcsi, 2008). miR‐146a has been shown to be a circulating miRNA that is increased in synovial tissues and fibroblasts in rheumatoid arthritis (RA) patients as a particularly important negative regulator of nuclear factor‐κB (NF‐κB) signaling (Murata et al., 2010; Stanczyk et al., 2008; Taganov, Boldin, Chang & Baltimore, 2006). miR‐146a has also received much attention in the field of OA research. miR‐146a was one of the first microRNAs discovered in cartilage that is associated with osteoarthritis. However, its role during OA pathogenesis is controversial with some studies suggesting a protective function while other studies indicating a destructive role in cartilage homeostasis. For example, expression of miR‐146a in chondrocytes has been proposed to contribute to OA pathogenesis by diminishing the response to TGF‐β (Li et al., 2012). A most recent study seemed to correlate with this hypothesis in vivo (Zhang et al., 2017). However, other study supports an opposite model in which miR‐146a plays a protective anti‐inflammatory role in OA (Gu et al., 2015; Li et al., 2011). Thus, the in vivo role of miR‐146a in OA pathogenesis is unclear.

Notch is a single‐pass transmembrane receptor protein, which is activated by ligands on adjacent cells. The Notch receptors, including four members in mammals (Notch 1–4), are activated by binding with a number of ligands. Upon ligand binding, the intracellular Notch domain is cleaved and translocated to the nucleus, where it regulates downstream target gene transcription. Notch1 receptor is highly expressed by articular chondrocytes located on the surface of mouse and human articular cartilage (Sassi et al., 2014). Notch signaling has been shown to be involved in cartilage homeostasis and OA development (Karlsson, Brantsing, Egell & Lindahl, 2008; Liu et al., 2015). However, its role is controversial with some studies showing a stimulatory effect (Hosaka et al., 2013), while others showing an inhibitory effect on OA pathogenesis (Mirando et al., 2013). Because Notch1 contains a miR‐146a‐binding site, we determined whether Notch1 was involved in miR‐146a regulation of OA in this study.

To study the functional role of miR‐146a in joint homeostasis in vivo, we analyzed genetic models of both miR‐146a loss of function and gain of function in this study. Mice with targeted miR‐146a deletion represent one of the first genetic animal models with NF‐κB‐driven low‐grade inflammation that develops spontaneously with aging. We analyzed miR‐146a gene knockout mice to determine the effect of such low‐grade inflammation on cartilage homeostasis during aging. We generated miR‐146a cartilage‐specific overexpression transgenic mice by crossing floxed miR‐146a transgenic mice with Col2a1‐Cre mice. These mice were characterized during both aging‐associated OA and post‐traumatic OA induced by destabilization of the medial meniscus model (DMM). We provided evidence that deficiency of miR‐146a led to the early onset and progression of OA. Conversely, overexpression of miR‐146a protected against both aging‐ and trauma‐associated OA. This protective mechanism appears to involve inhibition of Notch1, IL‐6, and IL‐1 signaling by miR‐146a.

## 2 RESULTS

### 2.1 miR‐146a, mechanoresponsive and anti‐inflammation, is downregulated in human OA lesions

**Figure 1:**
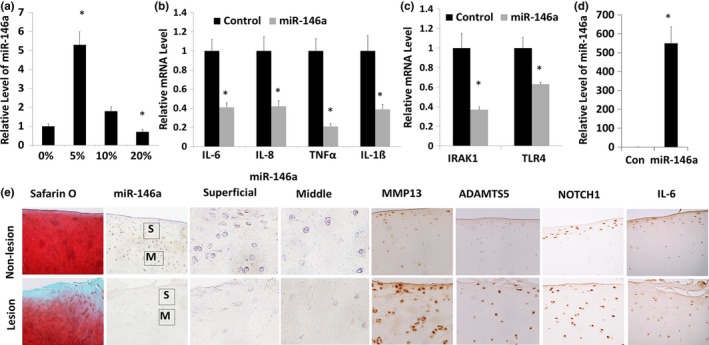
Mechanically responsive miR‐146a downregulated inflammatory cytokines and is expressed at a lower level in OA cartilage. (a) Regulation of miR‐146a expression levels by mechanical loading. Human articular chondrocytes isolated from human cartilage obtained during joint replacement surgery. Chondrocytes were cultured in 3D collagen sponges and cyclic loaded to induce 5%, 10%, or 20% deformation of the sponge at 1 Hz, for 24 hr. Expression levels of miR‐146a were quantified by real‐time PCR. Data = means ± *SD*s. *p < .01 compared to nonload. Results are representative of analyses of three patients. (b, c) miR‐146a inhibits the expression of inflammatory cytokines. miR‐146a mimic was transfected into human articular chondrocytes, and RNA was isolated at 48 hr post‐transfection. The expression levels of miR‐146a, IL‐1β, IL‐6, IL‐8, TNFα, IRAK1, and TLR‐4 were quantified by real‐time RT–PCR. Data = means ± *SD*s. (*n* = 6), *p < .05. (d) The expression level of miR‐146a was evaluated by miR‐146a miRNA assay to confirm transfection efficiency. (*n* = 6), *p < .05. SnoRNA U6 was used as an endogenous control for miRNA expression, while 18s ribosome RNA was used for normalization of mRNA expression. Data = means ± *SD*s. *p < .05. Results are representative of analyses of three patients. (e) In situ hybridization analysis showed miR‐146a is expressed at a lower level in damaged OA cartilage. OA cartilage isolated from the loaded area exhibited OA changes. Nonweight‐bearing area with normal looking cartilage from same patient was used as control. S, superficial zone of the articular cartilage. M, middle zone of articular cartilage. Proteoglycan loss was shown by Safranin O staining of human cartilage. The protein expressions of MMP13, ADAMTS5, Notch1, and Il‐6 were detected by immunohistochemistry analysis. Results are representative of analyses of six patients

To determine whether miR‐146a is mechanoresponsive in human articular chondrocytes, we performed real‐time PCR using human chondrocytes cultured in a 3D collagen sponge subjected to cyclic loading. The expression level of miR‐146a was increased 5.1‐fold in response to 5% cyclic matrix deformation for 24 hr. However, 10% cyclic matrix deformation did not change miR‐146a level significantly, while 20% cyclic matrix deformation significantly inhibited miR‐146a level in human chondrocytes. Therefore, miR‐146a level was increased by mild mechanical loading and inhibited by supra‐physiological loading (Figure 1a).

To determine whether miR‐146a is associated with expression of pro‐inflammatory cytokines in human articular chondrocytes, we performed RT–PCR analysis of inflammatory gene expression after transfecting miR‐146a in human chondrocytes. The expression of *IL‐1*β, *IL‐6*,* IL‐8*, and *TNF*α were downregulated in miR‐146a‐transfected chondrocytes (Figure 1b), along with miR‐146a known targets *IRAK1* and *TLR4* (Figure 1c). The expression level of miR‐146a was evaluated by miR‐146a miRNA assay to confirm successful transfection (Figure 1d). Thus, miR‐146a suppresses important inflammatory molecules in chondrocytes.

To determine whether miR‐146a is involved in OA pathogenesis, we performed in situ hybridization of miR‐146a and compared its expression level between the nonlesion and lesion areas of articular cartilage from human OA patients. The OA cartilage lesion exhibits significant proteoglycan loss as shown by reduced Safranin O staining (Figures 1e and S1) and significantly lower miR‐146a expression in both superficial and middle zones. In the nonlesion area, miR‐146a is localized in the cytoplasm of chondrocytes throughout the whole thickness of cartilage (Figure 1e). Expression of matrix‐degrading matrix enzymes MMP13 and ADAM metallopeptidase with thrombospondin type 1 motif 5 (ADAMTS5) were significantly increased in OA lesion compared with nonlesion region (Figure 1e). The expression of Notch1 and IL‐6 were also upregulated in OA cartilage lesion (Figure 1e).

### 2.2 Deficiency of miR‐146a causes OA‐like pathology in mice

**Figure 2:**
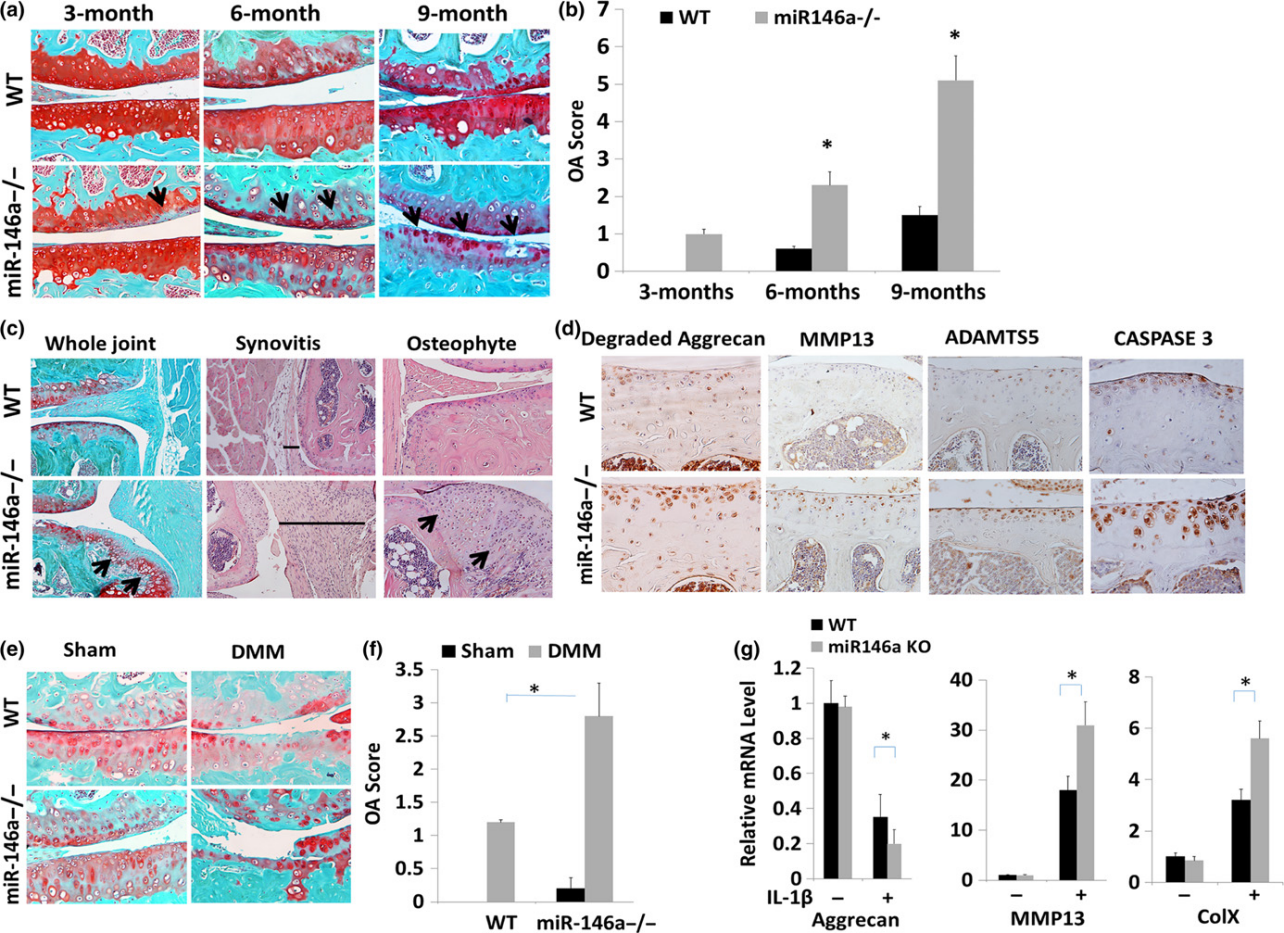
Loss of miR‐146a causes early onset of OA changes during aging and exacerbates cartilage degradation in surgery‐induced OA development. (a) Safranin O staining of the knee joint in 3‐month‐old mice, 6‐month‐old mice, and 9‐month‐old mice. miR‐146a deletion caused an early onset of proteoglycan loss and structure damage as indicated by arrow. (b) Cartilage destruction was quantified, and the summed OA scores were significantly increased in *miR‐146a* ^−/−^ mice. Data are expressed as means ± *SD*s. (*n* = 8), *p < .05. (c) Representative H&E or Safranin O and fast green staining of the knee joint in 9‐month‐old mice sections of WT and *miR‐146a* ^−/−^ mice, demonstrating synovitis (lines) and osteophyte development on the anterior tibial plateau (arrows). (d) Cartilage sections from 9‐month‐old WT and *miR‐146a* ^−/−^ mice were immunostained for degraded Aggrecan (neoepitope DIPEN), Mmp13, Adamts5, as well as caspase‐3. (e) Safranin O staining of the knee joint in 8‐week‐old DMM‐operated WT and miR‐146a mice. (f) Cartilage destruction was quantified, and the summed OA scores were significantly increased in *miR‐146a* ^−/−^ mice. Data are expressed as means ± *SD*s. (*n* = 10), *p < .05. (g). Deficiency of miR‐146a induces IL‐1β induction of Mmp13 and Col X. Primary mouse articular chondrocytes were isolated from 5‐day‐old WT and *miR‐146a* ^−/−^ mice and treated with or without IL‐1β for 24 hr. mRNA levels of *Aggrecan*,* Mmp13*, and *ColX* were detected by real‐time PCR analysis. Data = means ± *SD*s. (*n* = 6), *p < .05. SnoRNA U6 was used as an endogenous control for miRNA expression, while 18s ribosome RNA was used for normalization of mRNA expression

To determine whether the loss of miR‐146a leads to OA pathogenesis, we characterized the *miR‐146a* ^−/−^ mouse strain, *miR‐146a* ^−/−^ **(**B6.Cg‐*Mir146* ^*tm1.1Bal*^/J; Boldin et al., 2011). The level of miR146 was greatly reduced in cartilage of *miR‐146a* ^−/−^ mice, although the tissue structure of knee joints including articular cartilage, menisci, and ligaments appeared to be normal in *miR‐146a* ^−/−^ mice from birth until 2 months old (data not shown). However, knee joints from 3‐month‐old mice showed reduced Safranin O staining at the surface of femoral condyles and tibial plateaus, indicative of proteoglycan loss (Figure 2a). More severe proteoglycan loss and fibrillation on articular surfaces were observed in 6‐month‐old *miR‐146a* ^−/−^ mice. Overt cartilage degradation was apparent as more severe proteoglycan loss and structural cartilage defects were observed in 9‐month‐old *miR‐146a* ^−/−^ mice (Figure 2a). Osteoarthritis scores were significantly higher in 6‐and 9‐month‐old *miR‐146a* ^−/−^ mice compared with age‐matched wild‐type mice (Figure 2b). Furthermore, joint tissue exhibited osteophyte formation and synovial hyperplasia in 9‐month‐old *miR‐146a* ^−/−^ mice (Figure 2c). Notably, expression of matrix‐degrading enzymes ADAMTS5 and MMP13 as well as caspase‐3 were upregulated in 9‐month‐old *miR‐146a* ^−/−^ mice (Figure 2d). Consistent with this, cartilage‐specific proteoglycan fragments cleaved by ADAMTs were also increased (Figure 2d). We next determined whether loss of miR‐146a affects post‐traumatic osteoarthritis induced by destabilization of medial meniscus (DMM). Safranin O/fast green staining showed that deficiency of miR‐146a significantly exacerbates cartilage destruction with significant increase in summed OA score at 8 weeks post‐DMM surgery (Figure 2e,f). The results collectively suggest that genetic deletion of miR‐146a promotes OA and PTOA pathogenesis in mice.

To determine whether miR‐146a is associated with OA pathology, we further examined mRNA levels of anabolic and catabolic genes in primary cultures of mouse articular chondrocytes isolated from *miR‐146a* ^−/−^ and WT mice. We did not find significant differences in the expression of *Aggrecan*,* Mmp13* or *Col X* in miR‐146a‐deficient chondrocytes compared with WT control. However, following treatment with IL‐1β, a pro‐inflammatory cytokine that promotes catabolism in articular cartilage (Kapoor, Martel‐Pelletier, Lajeunesse, Pelletier & Fahmi, 2011), mRNA level of *Mmp13* and *Col X* was significantly increased, while *Aggrecan* was decreased in miR‐146a‐deficient chondrocytes in comparison with WT chondrocytes (Figure 2g). This suggests that miR‐146a modulates chondrocyte anabolic and catabolic activities through affecting cytokine regulation of gene expression.

### 2.3 miR‐146a overexpression in cartilage tissue inhibits OA and PTOA pathogenesis

**Figure 3:**
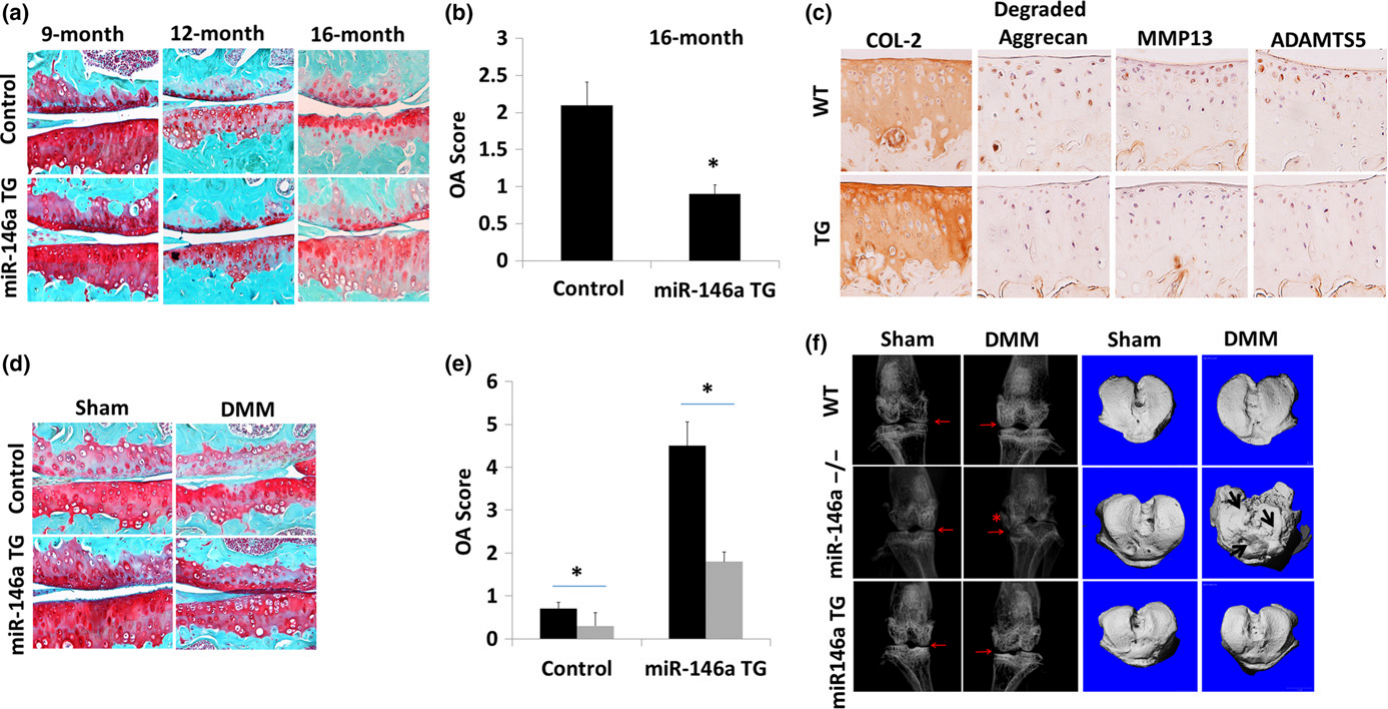
Chondrocyte‐specific miR‐146a overexpression in cartilage inhibited cartilage destruction. (a) Spontaneous cartilage destruction was determined by Safranin O staining in 9‐, 12‐, and 16‐month‐old miR‐146a TG mice or its age‐ and sex‐matched littermate control mice. Sixteen‐month‐old miR‐146a TG mice exhibited less OA‐associated phenotypes (proteoglycan loss and structure damage) than the control mice. (b) Cartilage sections from 16‐month‐old WT and *miR‐146a* ^−/−^ mice were immunostained for degraded Aggrecan, MMP13, Aamts5, Ihh, and Col II. (c) Cartilage destruction was quantified and the summed OA scores were significantly lower in miR‐146aTG mice. Data are expressed as means ± *SD*s. (*n* = 10), *p < .05. (d) miR‐146a overexpression in cartilage delayed surgery‐induced OA development. Surgical OA was induced by DMM surgery in 8‐week‐old Col2a1‐Cre; WT or Col2a1‐miR‐146a TG mice or *miR‐146a* ^−/−^ mice. The knee joints were harvested 8 weeks after surgery and stained with Safranin O/fast green. Proteoglycan loss was inhibited in miR‐146a TG mice. (e) The summed OA scores was significantly reduced in miR‐146a TG mice in comparison with surgery legs from their littermate control. Data are expressed as means ± *SD*s. (*n* = 10), *p < .05. (f) Surgical OA was induced by DMM surgery in 8‐week‐old Col2a1‐Cre; WT or Col2a1‐miR‐146a TG mice or *miR‐146a* ^−/−^ mice and the knee joints were collected at 12 weeks after surgery. The left panel of (f) is the images from X‐ray analysis to assess joint space as shown by red arrow. Joint space was well maintained in the DMM‐operated knee in miR‐146a TG mice but narrow and severely deformed with osteophyte formation (star) in *miR‐146a* ^−/−^ mice. The right panel of F showed the images from low power micro‐CT analysis which was performed to evaluate the cartilage degeneration. We distinguished cartilage tissue from non‐cartilage tissue by segmenting the grayscale images at 263 (lower threshold) to 1,000 (upper threshold). Cartilage surface is smooth in the DMM‐operated knee in Col2a1‐miR‐146a TG mice, but very rough in the DMM‐operated knee in *miR‐146a* ^−/−^ mice

To determine whether miR‐146a is sufficient for inhibiting OA, we generated miR‐146a‐floxed transgenic mice (Figure S2). After crossing these mice with Col2a1‐Cre transgenic mice, chondrocyte‐specific miR‐146a‐overexpressing transgenic mice were then generated (miR‐146aTG for short). Among multiple transgenic lines, we selected a miR‐146aTG line in which miR‐146a is overexpressed about 4.2‐fold in cartilage (Figure S2c), which mimics the extent of miR‐146a stimulation by moderate mechanical loading (5%) induced cyclic matrix deformation. In littermate control WT mice, articular cartilage was relatively normal at 9 months and 12 months while developing cartilage surface fissuring at 16 months (Figure 3a). miR‐146aTG mice exhibited a significant reduction in OA cartilage degeneration score compared littermates control mice at 16 months (Figure 3a,c). Immunohistochemistry analysis revealed that the expression of COL II was upregulated in miR‐146aTG mice, while the expression of the neoepitope of AGGRECAN cleavage fragments was downregulated. Consistently, the catabolic genes ADAMTS5 and MMP13 were significantly downregulated (Figure 3b).

To determine whether overexpression of miR‐146a also affects PTOA progression, we performed DMM surgery to induce PTOA and examine the cartilage at 8 weeks post‐DMM surgery. miR‐146aTG mice exhibited significant inhibition of proteoglycan loss and surface fissuring in articular cartilage induced by DMM in comparison with control mice (Figure 3d). Furthermore, OA scores were significantly reduced in miR‐146aTG mice in comparison with control (Figure 3e). We examined the knee joint space of WT mice, miR‐146aTG mice, and *miR‐146a* ^−/−^ mice 12 weeks after DMM surgery by X‐ray. While *miR‐146a* ^−/−^ mice exhibited a deformed and narrowed joint space (arrow) and an osteophyte (star) of the knee joints, they were protected in miR‐146aTG mice (Figure 3f). We also performed micro‐CT reconstruction to visualize cartilage surface smoothness. The surgery leg in miR‐146a^−/−^ mice exhibited a rough articular surface and also osteophyte formation compared with WT and miR‐146aTG mice (Figure 3f). These results collectively suggest that while miR‐146a deficiency promotes OA pathogenesis, overexpression of miR‐146a protects cartilage from degeneration.

### 2.4 Notch1 is a direct target of miR‐146a and mediates OA pathogenesis

**Figure 4:**
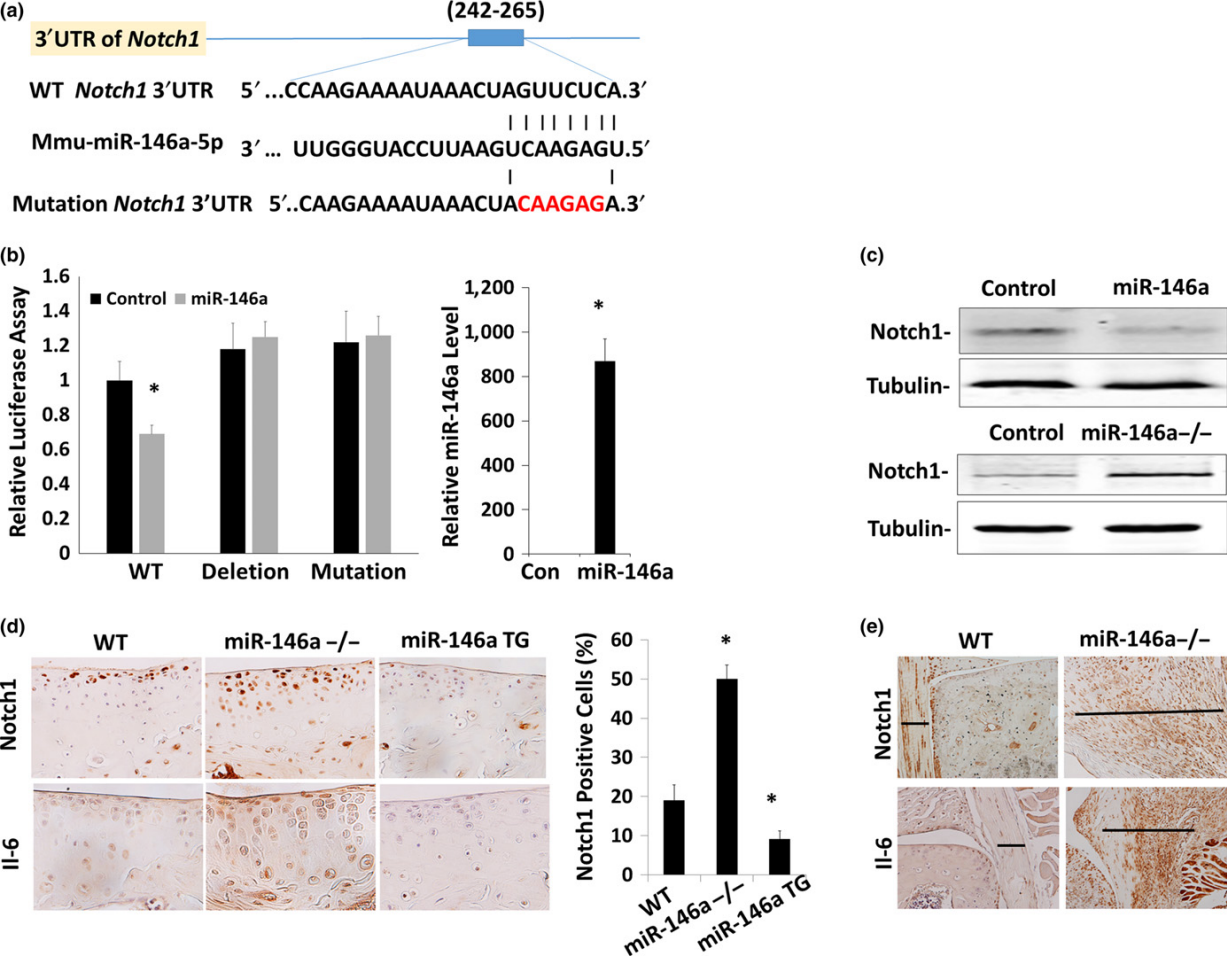
Notch1 is a direct target of miR‐146a. (a) Diagram of predicted miR‐146a sequential pairing with a putative target region (miR‐146a‐binding site) in the 3′‐UTR of *Notch1* mRNA. The 5′ end of miR‐146a contains a base pair sequence complementary to the putative seed site in *Notch1* mRNA. The mutant plasmid was mutated six base pairs of the seed region of the miR‐146a‐binding site to their complementary sequence. The whole binding sequence was deleted for the deletion plasmid. (b) ATDC5 cells were cotransfected with a reporter carrying a WT, mutant, or deletion of Notch1 3′UTR along with miR‐146a or its control mimics and were analyzed by dual‐luciferase assay. Luciferase activity was significantly inhibited in co‐transfection of miR‐146a with psiCHECK™‐2‐*Notch1* 3′ UTR vector. The luciferase activities were not affected in co‐transfection of miR‐146a with mutation and deletion plasmids. The ratio of firefly vs. Renilla luciferase is the relative value of reporter activities. The expression level of miR‐146a was evaluated by miR‐146a miRNA assay to confirm transfection efficiency. Data are expressed as means ± *SD*s. (*n* = 3), *p < .05. (c) Western blot analysis of protein expression of Notch1 and tubulin. It is representative from three independent Western blots analysis. Total cell lysates were extracted 48 hr post‐transfection or isolated from primary chondrocytes from *miR‐146a* ^−/−^ mice and the littermate controls (Control). Equal amount of protein was used for Western blot analysis with an antibody against Notch1, and tubulin was used as a loading control. (d and e) Cartilage sections (d) and synovium (e) from 9‐month‐old WT,* miR‐146a* ^−/−^, and miR‐146a TG mice were immunostained for Notch1 and IL‐6. Notch1‐positive cell were counted and divided by total cells showing changes in Notch1 expression in articular cartilage. Nine sections from three animals of each genotype were counted. *p < .05. The lines indicated the thickness of synovium. The expression of Notch1 and IL‐6 was increased in *miR‐146a* ^−/−^ mice while decreased in *miR‐146a* TG mice compared to littermate control mice

To determine whether Notch1 is a direct miR‐146a target, the *Notch1* 3′‐UTR, which includes a putative miR‐146a‐binding site (Figure 4a), was cloned into 3′‐UTR of *Notch1* gene downstream of the luciferase gene in an expression vector driven by the SV‐40 promoter. Transfection of miR‐146a mimic significantly reduced the luciferase activity, but not when the binding site was either deleted (deletion) or mutated (mutation; Figure 4b). Western blot analysis indicated that Notch1 protein level was downregulated by miR‐146a transfection in chondrocytes and upregulated in primary chondrocytes from *miR‐146a* ^−/−^ mice in comparison with WT mice (Figure 4c). Therefore, miR‐146a inhibits Notch1 expression through targeting its binding site at 3′‐UTR of *Notch1* gene.

As we found that miR‐146a was downregulated while Notch1 was upregulated in human OA articular cartilage lesions (Figure 1e), we analyzed the distribution of Notch1 in *miR‐146a* ^−/−^ and miR‐146aTG mice. Immunohistochemistry analysis showed that Notch1 protein expression in articular cartilage was upregulated in *miR‐146a* ^−/−^ mice and downregulated in miR‐146aTG mice (Figure 4d). Furthermore, both Notch1 and IL‐6 were upregulated in articular cartilage (Figure 4d) and synovium (Figure 4e) of *miR‐146a* ^−/−^ mice. Taken together, these data indicate that miR‐146a directly inhibits Notch1 expression.

**Figure 5:**
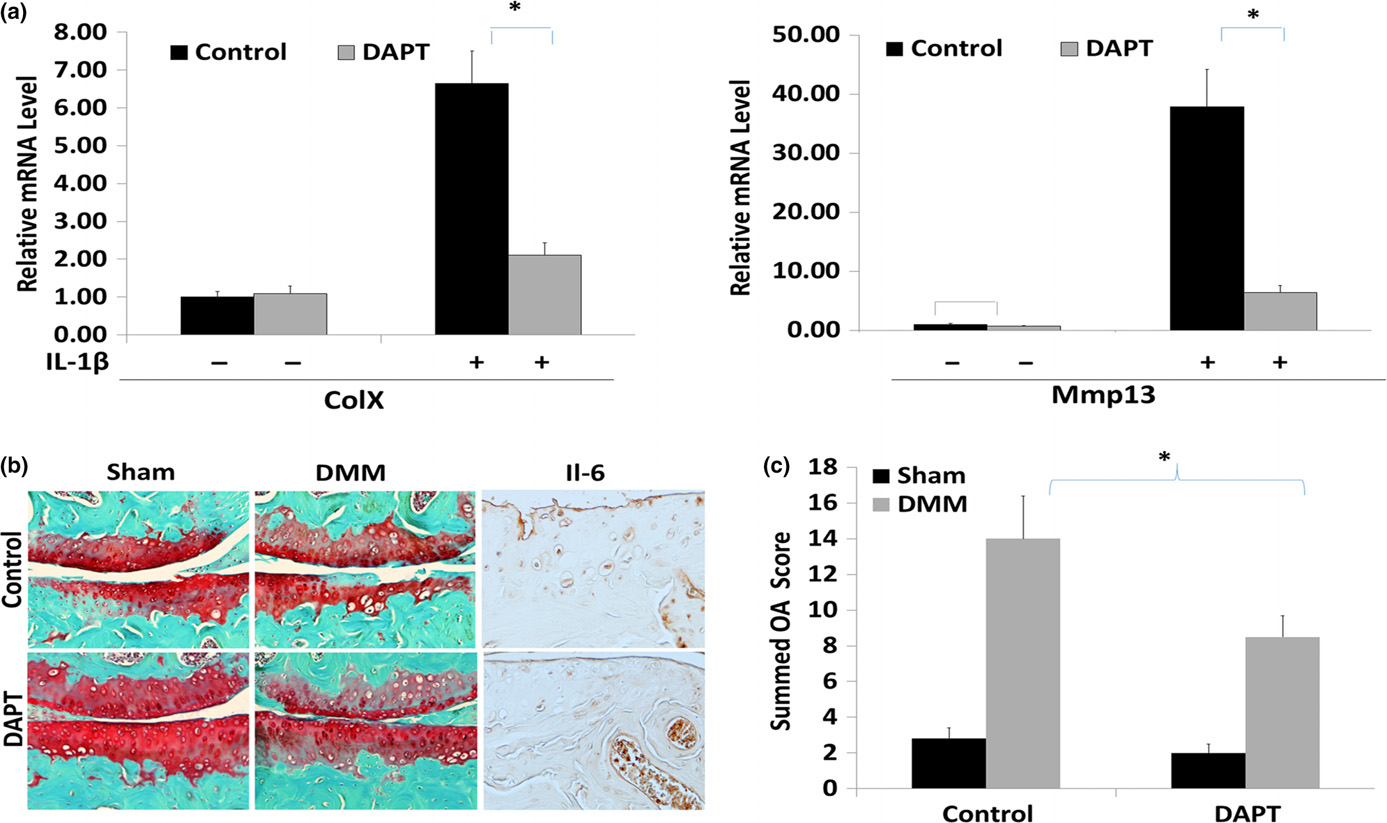
Inhibition of Notch signaling alleviates IL‐1β‐induced catabolic effects and DMM surgery‐induced OA in *miR‐146a* ^−/−^ mice. (a) Primary articular chondrocytes were isolated from *miR‐146a* ^−/−^ mice. Cells were pretreated with or without 10 nm DAPT for one hour and then added 10 ng/ml of IL‐1β for additional 24 hr. The expressions of *MMP13* and *ColX* were detected by real‐time PCR. Data are expressed as means ± *SD*s. (*n* = 3), *p < .05. (b) Intra‐articular injection of DAPT suppressed the cartilage degradation in DMM‐induced OA model in *miR‐146a* ^−/−^ mice compared with the vehicle‐injected knee joints. Cartilage sections were stained for Safranin O/fast green or immunostained with IL‐6. (c) Cartilage destruction was quantified, and the summed OA scores were significantly inhibited in DAPT‐injected legs. Data are expressed as means ± *SD*s. (*n* = 8), *p < .05

As IL‐6 and MMP‐13 were upregulated together with Notch1 in human OA cartilage lesions (Figure 1e), we determined whether Notch1 is directly associated with IL‐6 and MMP‐13 upregulation in *miR‐146a* ^−/−^ mice. We inhibited Notch1 using DAPT (N‐[N‐(3,5‐diflurophenylacetate)‐L‐alanyl]‐(S)‐phenylglycine t‐butyl ester), a specific pharmacological inhibitor of the Notch signaling pathway both in vitro and in vivo. Inhibiting Notch1 in chondrocytes suppressed expression of OA markers Col X and MMP‐13 induced by IL‐1β treatment, but did not suppress their basal level expression in the absence of IL‐1β (Figure 5a). Intra‐articular injection of DAPT suppressed DMM‐induced cartilage degeneration (Figure 5b,c) and inhibited upregulation of IL‐6 in *miR‐146a* ^−/−^ mice in vivo (Figure 5b). Thus, miR‐146a‐deficiency‐induced cartilage degeneration may be mediated by Notch1.

### 2.5 Restoring miR‐146a in miR‐146a‐deficient chondrocytes rescues the catabolic effects induced by IL‐1β or DMM

**Figure 6:**
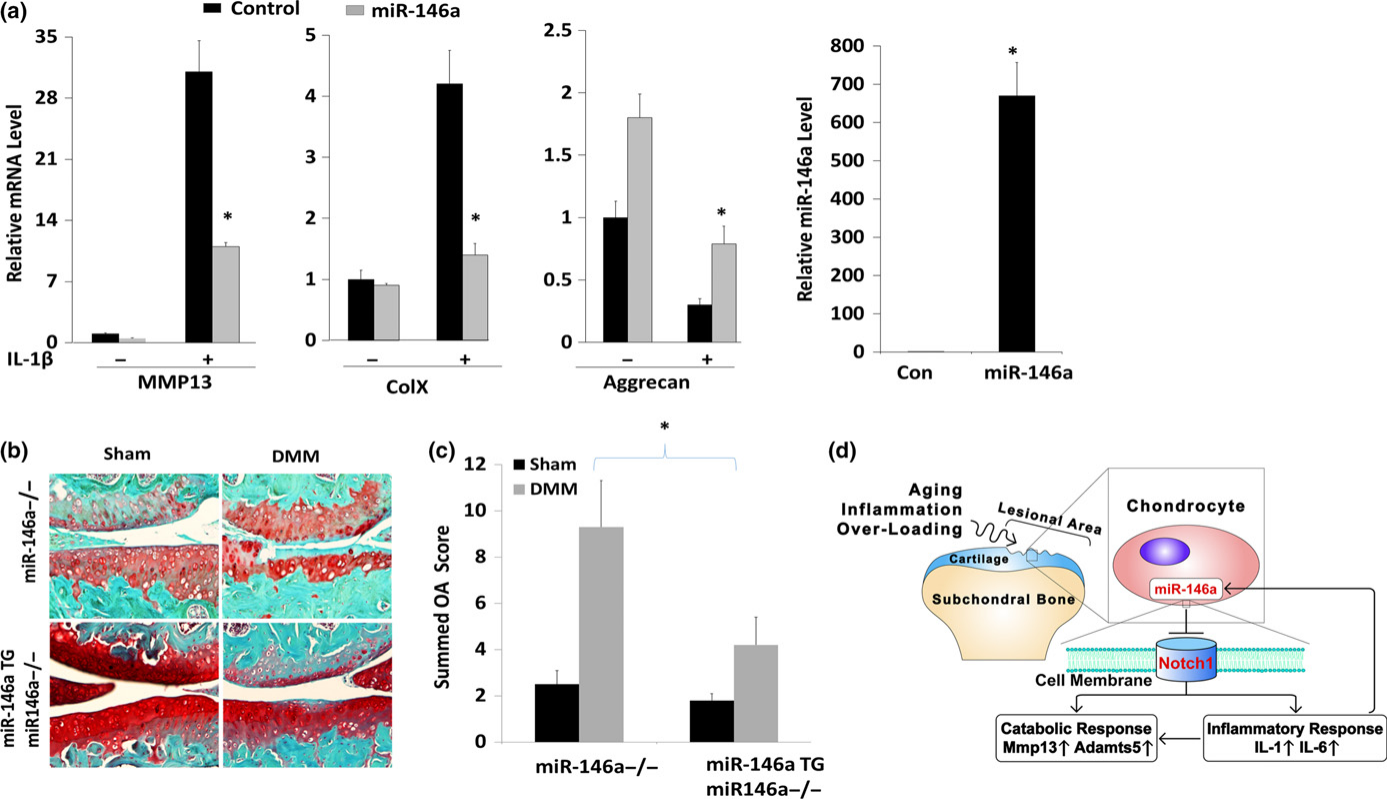
miR‐146a overexpression rescues IL‐1β‐induced catabolic effect or DMM‐induced OA. (a) miR‐146a overexpression inhibits IL‐1β‐induced catabolic effect in human articular chondrocytes. Chondrocytes were transfected with miR‐146a mimic or its negative control mimic for 24 hr and then treated with or without IL‐1β for 24 more hours. The mRNA levels of *Aggrecan*,* Mmp13*, and *Col X* were quantified by real‐time PCR analysis. The expression level of miR‐146a was evaluated by miR‐146a miRNA assay to confirm transfection efficiency. Values are presented as means ± *SD*s. Results are representative of analyses of three patients. *p < .05. (b) Safranin O staining of the knee joint in 8‐week‐old DMM‐operated Col2a1Cre;miR‐146a^f/+^; *miR‐146a* ^−/−^ mutant mice and the littermate control Col2a1Cre;WT; 146a^−/+^ mice. (c) Cartilage destruction was quantified, and the summed OA scores were significantly increased in *miR‐146a* ^−/−^ mice. Data are expressed as means ± *SD*s. (*n* = 5), *p < .05. (d) The diagram that we proposed in which miR‐146a protects the cartilage joint degradation by inhibiting the feedback loop of Notch1/IL‐6

OA occurs in *miR‐146a* ^−/−^ mice in which miR‐146a is deficient in all tissues. To determine whether restoration of miR‐146a expression in chondrogenic cells is sufficient for rescuing OA phenotype, we overexpressed miR‐146a both in vitro and in vivo. Transfection of miR‐146a mimic significantly inhibited IL‐1β stimulation of catabolic genes *MMP13* and *ColX* and rescued IL‐1β suppression of anabolic gene *Aggrecan* in human OA chondrocytes (Figure 6a).

To determine whether restoring miR‐146a in chondrogenic cells could rescue OA progression in *miR‐146a* ^−/−^ mice, we generated the Tg(Col2a1‐Cre;miR‐146a); *miR‐146a* ^−/−^ mice by crossing *miR‐146a* ^−/−^ mice with miR‐146aTG mice. In such transgenic mice of which miR‐146a expression was restored in chondrogenic cells, DMM‐induced cartilage degradation was suppressed in comparison with *miR‐146a* ^−/−^ mice (Figure 6b). Summed OA score confirmed quantitatively that restoring miR‐146a in cartilage significantly suppressed OA development in miR‐146‐deficient mice (Figure 6c).

## 3 DISCUSSION

While genetics accounts for about 50% of OA pathogenesis, the other 50% is due to other factors including epigenetics (Im & Choi, 2013). It becomes clear that multiple environmental factors are critical to epigenetic regulation of OA. They include aging, inflammation, and joint mechanics (Loeser, Collins & Diekman, 2016). However, it is not clear how these diverse factors regulate epigenetics of OA. MicroRNAs are posttranscriptional regulators of gene expression that control a wide range of biological processes (Selbach et al., 2008). We demonstrate in this study that miR‐146a may be a key factor that regulates OA pathogenesis by responding to aging, inflammation, and mechanical loading, thereby potentially linking these diverse OA‐driving factors. We were the first to identify miR‐146a as a mechanoresponsive microRNA along with miR‐365 through cytomechanical screening of microRNA microarray (Guan, Yang, Wei & Chen, 2011). Since then, multiple studies have supported the role of miR‐146a as a mechano‐miR (Huang, Crawford, Higuita‐Castro, Nana‐Sinkam & Ghadiali, 2012; Li et al., 2012). In this study, we further show that, while cyclic load of human articular chondrocytes in 3D culture in physiological range stimulated miR‐146a expression, mechanical overload inhibited miR‐146a expression. Such dichotomous effects of mechanical loading, which depend on its amplitude, have been observed in OA pathogenesis. While moderate exercise and weight loss have generally been shown to promote beneficial protective effects for osteoarthritic joints, altered joint loading—associated with obesity, misalignment, trauma, or joint instability—is a critical risk factor for joint degeneration (Cicuttini & Wluka, 2014). Thus, mechanical overload, such as what occur in joint cartilage during aging‐associated OA and injury‐induced PTOA, may exacerbate joint degeneration by inhibiting miR‐146a expression. Such hypothesis is supported by in vivo evidence that miR‐146a‐deficient mice exacerbate joint degeneration in both OA and PTOA in this study.

Despite the association with mechanical overloading and aging, OA is no longer considered a “wear and tear only” disease. Instead, inflammation has been proposed to play an important role in OA pathogenesis (Loeser et al., 2016; Rainbow, Ren & Zeng, 2012). Aging is associated with inflammation through acquisition of a senescence‐associated secretory phenotype (SASP) with IL‐6 and IL‐1 being the most prominent cytokines (Coppe, Desprez, Krtolica & Campisi, 2010). Furthermore, mechanical overloading induces translocation of NF‐κB into the nucleus, stimulation of NF‐κB signaling pathway, and IL‐6 transcription activation in chondrocytes (Marcu, Otero, Olivotto, Borzi & Goldring, 2010; Olivotto, Otero, Marcu & Goldring, 2015). Therefore, biochemical and biomechanical crosstalk among different tissue compartments likely plays an important role in the onset and progression of OA by initiation and maintenance of a persistent low‐grade pro‐inflammatory environment in the joint (Little & Hunter, 2013).

Our data suggest that miR‐146a could be such an important mediator of inflammation in the joint during OA. The mediator role of miR‐146a in OA pathogenesis is multifaceted. First, the expression of miR‐146a was stimulated by inflammatory factors such as interleukin 1 and tumor necrosis factor‐alpha (Sheedy & O'Neill, 2008). It was one of the first microRNAs associated with OA cartilage (Yamasaki et al., 2009). miR‐146a operates to fine‐tune inflammatory responses via a “negative regulatory loop” (Ma, Becker Buscaglia, Barker & Li, 2011). We show that miR‐146a expression is decreased in OA lesions in comparison with nonlesion areas in cartilage samples of OA patients. It correlates inversely with OA markers such as MMP‐13 and ADAMTS5 in the OA lesions. Yamasaki et al. observed that miR‐146a is stimulated by IL‐1 and expressed intensely in mild OA cartilage (Yamasaki et al., 2009). However, in moderate and severe OA samples, miR‐146a expression is significantly decreased, in parallel to the increase in MMP‐13 level (Yamasaki et al., 2009). These evidences suggest that miR‐146a is stimulated by the increase in IL‐1 to counteract MMP‐13 upregulation and cartilage degeneration during OA pathogenesis.

We tested this hypothesis in this study. Depletion of miR‐146a in vivo results in an increase in OA hallmarks including MMP‐13, ADAMTS5, caspase‐3, Aggrecan degradation fragments, synovitis, osteophytes formation, and cartilage degeneration. miR‐146a gene knockout exacerbates joint degeneration in both aging‐associated OA and trauma‐induced PTOA mouse models. We further demonstrate that miR‐146a chondrogenic overexpressing mice are resistant to both aging‐ and trauma‐induced joint degeneration. These observations suggest that miR‐146a is an anti‐inflammatory microRNA that protects joints from degeneration associated with aging or trauma. This conclusion is consistent with previous studies that demonstrate an association of miR‐146a with anti‐inflammation phenotypes such as reduced pain in the joints (Gu et al., 2015; Li et al., 2011). However, we did not observe that miR‐146a gene knockout protects cartilage from degeneration in 12‐month‐old mice, as reported by a recent publication (Zhang et al., 2017).

We found that miR‐146a modulates chondrocyte inflammation by inhibiting expression of SASP cytokines including IL‐1β, IL‐6, TNFα, and IL‐8 and by modulating IL‐1β‐induced catabolic effects in chondrocytes. However, miR‐146a does not seem to affect expression of extracellular matrix genes in articular chondrocytes in the absence of inflammatory conditions. As miR‐146a affects cartilage homeostasis through regulating cytokine expression and signaling, it may explain why joint degenerative phenotypes manifest in miR‐146a‐deficient mice only during postyoung adulthood or in pro‐inflammatory environment induced by traumatic injuries.

We provide experimental evidence, both in vitro and in vivo, that Notch1 is closely associated with miR‐146a regulation of inflammatory cytokines and OA pathogenesis. Notch1 is a direct target of miR‐146a via interaction of its seeding site in the 3′ UTR. miR‐146a deficiency results in an increase in Notch1 and IL‐6 expression in articular cartilage and synovium, while miR‐146a overexpression results in their decrease in cartilage in vivo. Inhibition of Notch signaling using a specific inhibitor DAPT suppresses IL‐1β stimulation of OA‐associated genes in vitro and rescued joint cartilage degeneration in miR‐146a‐deficiency mice in vivo. Thus, miR‐146a inhibits OA pathogenesis possibly by targeting Notch1 that promotes inflammatory cytokine production and catabolism in the joint. The involvement of Notch1 in inflammation is consistent with a previous observation that Notch1 deficiency results in decreased inflammation, IL‐6 content, and downregulation of inflammatory responses (Quillard & Charreau, 2013). Furthermore, Notch pathway is highly activated in mouse and human joint tissue during post‐traumatic OA. Sustained Notch activation in adult joint cartilage contributes to a severe, early, and progressive OA‐like pathology (Hosaka et al., 2013; Liu et al., 2015), while temporary suppression of Notch signaling leads to the delayed progression of OA in murine joints (Hosaka et al., 2013).

Taken together, we propose that miR‐146a protects joint cartilage from degeneration through inhibiting the feedback loop of Notch1/IL‐6 during aging or trauma. miR‐146a may be a central mediator of several critical factors of OA pathogenesis including aging, inflammation, and mechanical overloading (Figure 6d). miR‐146a apparently functions through the Notch pathway to regulate inflammatory cytokines IL‐6 and IL‐1β and their catabolic effects on the joint tissues. miR‐146a content in joint cartilage appears to be critical to maintain cartilage homeostasis and prevent OA onset and progression. Restoration of miR‐146a in chondrogenic cells in the miR‐146a knockout mice is sufficient to rescue joint degeneration due to meniscus injury. This suggests that local supplement of miR‐146a in the joint may serve as a potential therapeutic for OA treatment to inhibit joint inflammation and degeneration and shift homeostasis away from catabolism during aging and injury.

## 4 EXPERIMENTAL PROCEDURES

### 4.1 Human cartilage tissue

Human cartilage tissue was aseptically collected at the time of total knee replacement surgery of OA patients. This study was performed with ethics committee approval, and all patients provided informed consent. Osteoarthritis was diagnosed according to the American Rheumatism Association Criteria for OA. Grade II and Grade III OA patients ages ranged from 59 to 67 years (62.7 ± 2.4, *n* = 13; female). Clinical data were carefully reviewed to exclude any forms of secondary OA and inflammatory joint diseases such as rheumatoid arthritis. The cartilage slices were observed under the microscope, and osteoarthritic damage score was assessed individually by three people. Human knee cartilage tissues were fixed and processed as described previously (Yang et al., 2016). Articular cartilage tissue sections were then stained with Safranin O/fast green or hematoxylin and eosin (H&E) or analyzed by immunohistochemistry. Primary human chondrocytes were isolated from human articular cartilage and were seeded into 6‐well plates for IL‐1β treatment, transfection, or seeded into 3D collagen sponges and subjected to 5%, 10%, and 20% elongation, 1 Hz cyclic loading for 24 hr as described previously (Guan et al., 2011; Yang et al., 2016).

### 4.2 Transgenic mice

All animal experiments were performed according to protocols approved by IACUC at Rhode Island Hospital. The *miR‐146a* **‐**deficient mouse strain, *miR‐146a* ^−/−^ **(**B6.Cg‐*Mir146* ^*tm1.1Bal*^/J) and wild‐type C57BL/6J (WT) mice, and Col2a1‐Cre mice [Tg (Col2a1‐cre)10Amc transgenic mice] (species: Mus musculus) (Sakai et al., 2001) were obtained from Jackson laboratory. We established colonies of miR‐146a‐floxed transgenic mice miR‐146a [Tg[B6.Cg‐Tg(CAG‐miR‐146a)1Chq/Bru] in the laboratory. miR‐146a^fl/+^ mice have been backcrossed with WT (C57BL/6J) mice for over seven generations to maintain in a C57BL/6J background. Using the strategy described previously to achieve cartilage‐specific overexpression of miRNA (de Vos et al., 2017), they were bred with Col2a1‐Cre mice to generate miR‐146aTG mice Col2a1Cre;miR‐146a [B6.Cg‐Tg(Col2a1‐Cre;miR‐146afl]. Genomic DNA isolated from the tail was analyzed by PCR using the primers used for confirmation of proper homologous recombination and genotyping are listed in Table S1. The age‐ and sex‐matched littermate control mice were used as the control mice. The *miR‐146a* ^−/−^ mice and wild‐type littermates (*n* = 10) were generated from the cross of *miR‐146a* ^−/+^ mice maintained on a C57BL/6 background. Col2a1Cre;miR‐146a^f/+^; *miR‐146a* ^−/−^ mutant [Tg(Col2a1‐Cre;miR‐146a); *miR‐146a* ^−/−^] mice were generated from breeding Col2a1Cre;WT or miR‐146a^f/+^;WT with *miR‐146a* ^−/−^ then further crossed to generate Col2a1Cre;miR‐146a^f/+^; *miR‐146a* ^−/−^ mutant mice, and the littermates of *miR‐146a* ^−/−^ mice were used as the control mice.

### 4.3 Surgical induction of OA by destabilization of the medial meniscus

All studies were performed with approval of the Institutional Animal Care and Use Committee. Knee joint instability was induced surgically in age‐matched male mice with desired genotype. At 8 weeks of age, 10 mice per group were anesthetized and instability of the right knee joint was induced by transection of the anterior attachment of the medial meniscus to the tibial plateau. Left knee joints were left intact and are termed “left unoperated control joints” (Botter et al., 2009; Glasson, Blanchet & Morris, 2007). For each intra‐articular administration of DAPT in *miR‐146a* ^−/−^ mice (Hosaka et al., 2013), we injected 5 μl of 5 μm DAPT solution, which was prepared by diluting 50 mm DAPT in dimethyl sulfoxide (DMSO) with injectable normal saline at 1:10,000, and 10 μl of DMSO diluted with normal saline (1:10,000) as the control. We performed intra‐articular administration of DAPT right after surgery and then once a week for 7 weeks after the surgical induction.

### 4.4 Radiographic imaging

X‐ray images of the entire knees, femurs, and tibiae were obtained immediately after euthanasia using a Faxitron X‐ray system (Wheeling) according to the manufacturers’ instructions to assess possible changes in the joint space. Low power micro‐CT analysis was used to characterize the joint cartilage surface. Specimens for microcomputed tomography (micro‐CT) were scanned using a SCANCO Medical AG micro‐CT scanner at 45kVp and 88 μA. The specimens were immobilized using cotton gauze and scanned to produce a voxel size of 6 μm. To study cartilage on the tibial surface, the outline of the tibial epiphysis was manually selected to exclude the meniscus. To distinguish calcified tissue from noncalcified tissue, grayscale images were uniformly segmented at 263 (lower threshold) and 1,000 (upper threshold) with default Gauss Sigma and Support settings. Three‐dimensional CT images of the tibial plateau were characterized further by three‐dimensional reconstruction, which were generated and measured using microview software (GE).

### 4.5 Histopathologic assessment

Mice were euthanized, and knee joints were harvested from 3‐, 6‐, and 9‐month‐old male miR‐146a^−/−^ mice or 9‐, 12‐, and 16‐month‐old male Col2a1‐Cre; miR‐146a and their age‐ and sex‐matched control mice. The knee joints were fixed, decalcified, and then stained with Safranin O/fast green or hematoxylin and eosin processed as described previously (Chen et al., 2016). Images are captured at 40× magnification with a Nikon E800 microscope (Melville, NY). Cartilage degeneration in articular cartilage surfaces was evaluated by two observers following the recommended semiquantitative scoring system in a blinded fashion (Glasson, Chambers, Van Den Berg & Little, 2010). Briefly, for histologic scoring of OA in the mouse, we used 0 to 6 scoring system to all four quadrants and through multiple sections through the joint. The OA severity is expressed as summed scores which combined the maximum score for the entire joint for medial femoral condyle, medial tibial plateau, lateral femoral condyle, and lateral tibial plateau. At least four sections per sample were analyzed microscopically and scored using previously reported semiquantitative scoring systems (Glasson et al., 2010).

### 4.6 Primary culture of chondrocytes and treatment

Mouse articular chondrocytes were isolated from femoral condyles and tibial plateaus of 5‐day‐old WT, miR‐146aTG and *miR‐146a* ^−/−^ mice, as described previously by digestion with collagenase D (Chu et al., 2016). We performed monolayer culture of primary articular chondrocytes with or without DAPT (Sigma, St. Louis, MO) in DMEM containing 10% (vol/vol) FBS, 10 mm β‐glycerophosphate, and 10 μg/ml ascorbic acid. Chondrocytes were treated with recombinant human IL‐1β (Roche, Branchburg, NJ) for the indicated concentration and periods of time. Human miR‐146a miRIDIAN microRNA mimics and hairpin inhibitor were obtained from Dharmacon (Lafayette, CO, USA). Human articular chondrocytes were transfected with each oligonucleotide using Lipofectamine 3000 (Life Technologies, Grand Island, NY). Forty‐eight hours after transfection, cells were harvested and subjected to total RNA and protein extraction.

### 4.7 Quantification of mRNA and miRNA

Total RNA in chondrocytes was extracted and quantified as described (Guan, Yang, Yang, Charbonneau & Chen, 2014; Yang et al., 2016). miR‐146a expression level was quantified with the TaqMan microRNA assays specific for mature miR‐146a (Life Technologies). Briefly, 10 ng RNA was transcribed using TaqMan microRNA RT Kit (Life Technologies) and followed by real‐time PCR with TaqMan MicroRNA Assays. The ubiquitously expressed miRNA, snoRNA U6, was used as an endogenous control. The mRNA levels were quantified by real‐time PCR with the SYBR Green PCR Master Mix (Qiagen). 18S ribosomal RNA was used as an internal control gene to normalize the mRNA levels. Relative transcription levels were calculated as previously described (Guan et al., 2011; Yang et al., 2016). In situ hybridization of miR‐146a was performed according to the manufacturer's instructions. LNA TM detection probe of miR‐146a and a control probe were purchased from EXIQON Inc. (Woburn, MA). Sections were hybridized with 40 nM double‐DIG LNATM miR‐146a Probe for 1 hr at 55°C. Staining was performed using BCIP and nitroblue tetrazolium (Roche, Branchburg, NJ).

### 4.8 Construction of plasmids, site‐directed mutagenesis, and luciferase assay

To create the psiCHECK™‐2‐Notch1 3′ UTR vector (Notch1‐3′UTR), a fragment of the 3′ UTR of Notch1 gene including the predicted miR‐146a‐binding site was PCR‐amplified and cloned into the Xho I and Not I sites of the psiCHECK™‐2 vector (Promega, Madison, WI) using the primers as listed in Table S1. The mutation and deletion of the seeding sites 3′‐UTR luciferase reporter plasmids were also generated by site‐directed mutagenesis using the QuikChange site‐directed mutagenesis kit (Stratagene, La Jolla, CA) using the primers as listed in Table S1. In mutant plasmid, six base pairs of the miR‐146a‐binding site were mutated. The whole binding sequence was deleted in the deletion plasmid. All sequences of the amplified products were confirmed by DNA sequencing. *Notch1*‐3′UTR reporter gene construct was assayed by co‐transfection of psiCHECK™‐2‐*Notch1*‐3′ UTR, its mutant or deletion with 50 nmol miR‐146a mimic or control miRNA in ATDC5 cells were transiently transfected using Lipofectamine 3000 (Invitrogen, Carlsbad, CA) according to the manufacturer's instructions. Twenty‐four hours after transfection, cells were lysed, and firefly and Renilla luciferase activities were measured using the Dual‐Luciferase Reporter Assay System (Promega, Madison, WI) according to the manufacturer's protocol. Each experiment was repeated at least three times.

### 4.9 Immunohistochemistry

Immunohistochemistry was carried out using the Histostain‐Plus 3rd Gen IHC Detection Kit (Invitrogen, Carlsbad, CA). The sections of knee joints were de‐paraffinized and rehydrated through conventional methods. Endogenous peroxidase was blocked by treating the sections with 3% hydrogen peroxide in methanol for 30 min. The sections were digested by 100 mg/ml hyaluronidase (Sigma, St. Louis, MO) for 15 min. Nonspecific protein binding was blocked by incubation with a serum blocking solution. The sections were then immunostained using antibodies against ADAMTS‐5, NOTCH1, and COL II (Abcam, Cambridge, MA), AGGRECAN neoepitope antibody representing the neoepitope sequence (NITEGE) generated by aggrecanase‐mediated cleavage at Glu ^373^‐Ala^374^ of AGGRECAN core proteins (Novus biologicals), and IL‐6 (Invitrogen, Carlsbad, CA), respectively, at 4°C overnight. The negative control sections were incubated with isotype control (Abcam) in 0.01m PBS. Thereafter, the sections were treated sequentially with ready‐to‐use biotinylated secondary antibody and ready‐to‐use streptavidin–peroxidase conjugate, followed by standardized development in DAB chromogen. The sections were counterstained with ready‐to‐use hematoxylin (Invitrogen). Images are captured at 40× magnification with a Nikon E800 microscope (Melville, NY).

### 4.10 Western blot analysis

Total protein extracts of transfected chondrocytes or chondrocytes isolated from rib cage of *miR‐146a* ^−/−^ mice at age of P5 were prepared for Western blot analysis as previously described (Yang et al., 2016). The following antibodies were used for this study: anti‐NOTCH1 (Abcam); anti‐tubulin (Santa Cruz Biotechnology Inc., Santa Cruz, CA); and anti‐mouse‐IRDye800 and anti‐rabbit‐Alexa Fluor 680 (Molecular Probes, Eugene, OR).

### 4.11 Statistical analysis

For cell culture experiments, all results are derived from experiments being repeated independently three times. For quantitative RT–PCR (qRT–PCR), data expressed as relative fold changes, Student's *t* test, and ANOVA with post hoc tests were used for pairwise and multiple comparisons, respectively, after normal distribution was confirmed using the Shapiro–Wilk test. Data quantified based on the OARSI ordinal grading system were analyzed using nonparametric statistical methods. Statistical significance was accepted at *p* < .05 for all analyses.

## CONFLICT OF INTEREST

None declared.

## AUTHORS’ CONTRIBUTION

Y. Guan and Q. Chen contributed to the conception and design of the study; acquisition, analysis, or interpretation of the data; and drafting and submission of the manuscript. Y. Guan and X. Yang performed the in situ hybridization of miR‐146a on human cartilage and DMM surgery. J. Li generated 3′UTR of Notch1 luciferase reporter plasmid and performed luciferase assay. Y. Guan, S. Du, J. Ding, and Y. Zhang performed the analysis of Western blot, immunohistochemistry, and staining, and scored the cartilage section. S. Du and Y. Gao performed X‐ray and μCT analysis. K. Yang provided the reagents and plasmid. All authors have read and approved the final submitted manuscript.
