# Supplementary figures and images for: Evidence that miR‐146a attenuates aging‐ and trauma‐induced osteoarthritis by inhibiting Notch1, IL‐6, and IL‐1 mediated catabolism
Source: Aging Cell. 2018 Mar 24;17(3):e12752. doi: 10.1111/acel.12752 (PMC5946074; doi:10.1111/acel.12752)

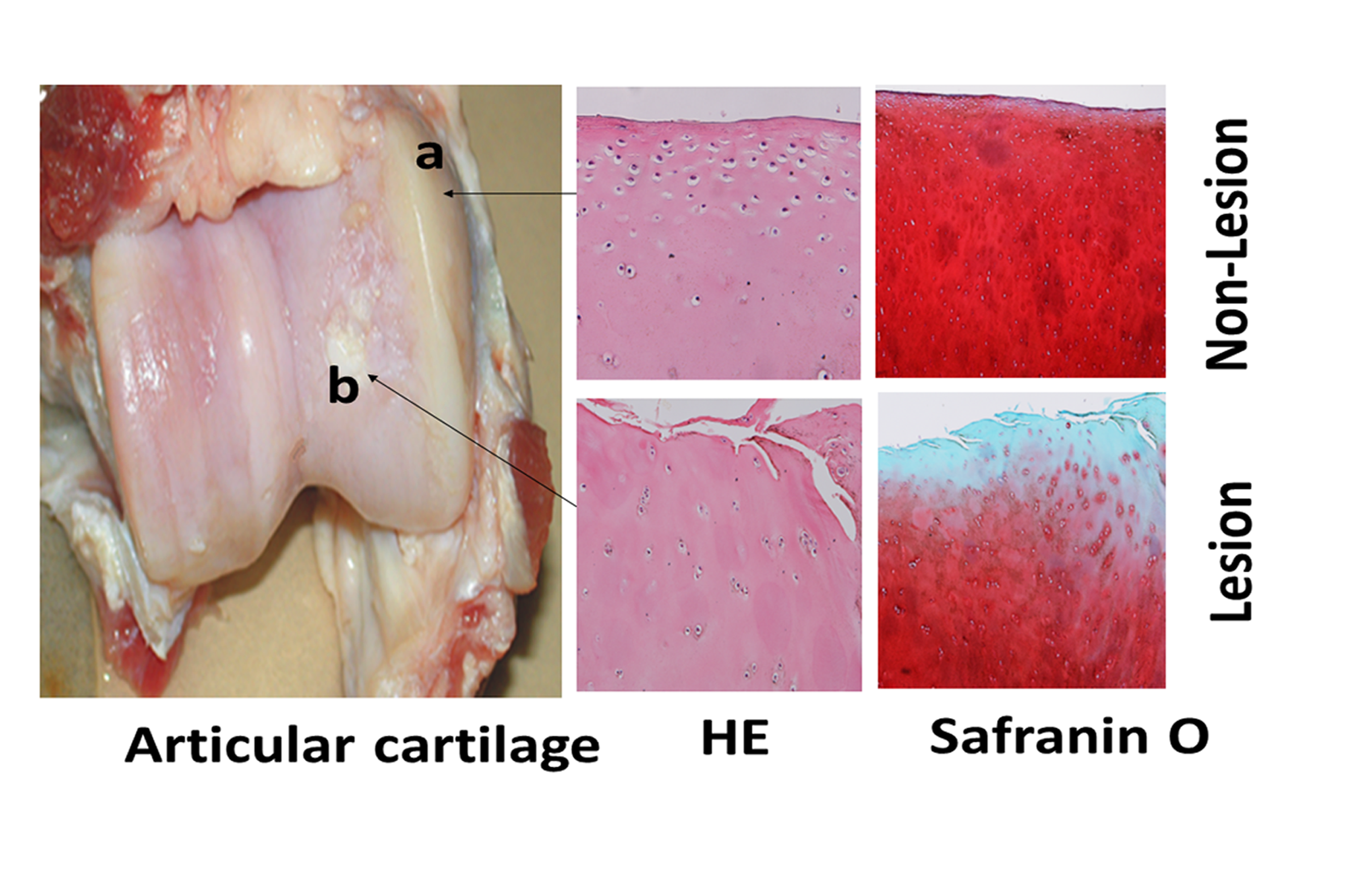

Supplement: Supplementary file 1 [file ACEL-17-e12752-s001.tif]

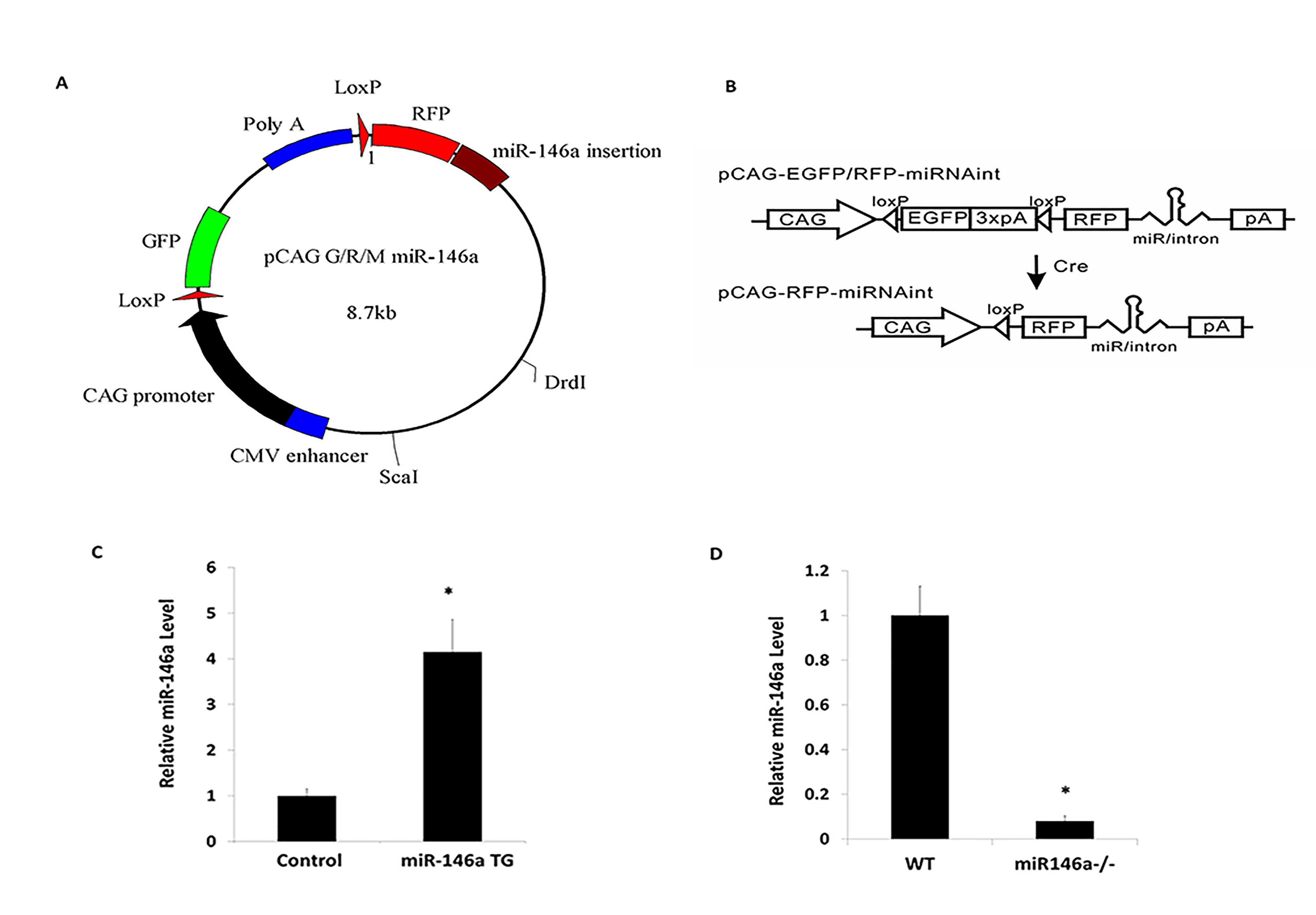

Supplement: Supplementary file 2 [file ACEL-17-e12752-s002.tif]
