# Supplementary material for: Evidence that miR‐146a attenuates aging‐ and trauma‐induced osteoarthritis by inhibiting Notch1, IL‐6, and IL‐1 mediated catabolism
Source: Aging Cell. 2018 Mar 24;17(3):e12752. doi: 10.1111/acel.12752 (PMC5946074; doi:10.1111/acel.12752)
Supplement: Supplementary file 3 [file ACEL-17-e12752-s003.docx]

**Supplemental Table 1.** Primers used in the study.

Primers used in the study

| Primers used | Sense 5’-3’ | Anti-Sense 5’-3’ |
| --- | --- | --- |
| \| miR-146a Transgenic mice \| \| --- \| \|  \| | AGGCCAGGTACCAGCTCTGAGAACTGAATTCCATGGGTTATATCAATGTCAGACCTGTG | TACCCGCTCGAGAGCTGAAGAACTGAATTTCACAGGTCTGACATTGATATAACCCATGG |
| genotyping of transgenic mice (miR-146a) | AGT GGG AGC GCG TGA TGA ACT TCG A - | CTG CTC CAC GAT GGT GTA GTC CTC GT |
| psiCHECK™-2 -*Notch1* -3’ UTR | CCGCTCGAGGAGATGTGGGATGCAGGACC | ATAAGAATGCGGCCGCTTCAAGGTCTGGGCGTGTTT |
| Mutation of *Notch1* 3’-UTR | GACCAGGAAAATCAAGGCTCTCTCTTGTAGTTTATTTTCTTGGAACAGATATGTTTTTCCCTAGTGC | GCACTAGGGAAAAACATATCTGTTCCAAGAAAATAAACTACAAGAGAGAGCCTTGATTTTCCTGGTC |
| Deletion of *Notch1* 3-’UTR | AGGAAAATCAAGGCTCTTAGTTTATTTTCTTGGAACAGATATGTTTTTC | GAAAAACATATCTGTTCCAAGAAAATAAACTAAGAGCCTTGATTTTCCT |
| *Mmp13* (mouse) | TGATGGACCTTCTGGTCTTCTGG | CATCCACATGGTTGGGAAGTTCT |
| *Aggrecan (*mouse) | GAAGACGACATCACCATCCCAG | CTGTCTTTGTCACCCACACATG |
| *Gapdh* (mouse) | TCACTGCCACCCAGAAGAC | TGTAGGCCATGAGGTCCAC |
